# Supplementary material for: Poly-drug use among female and male commercial sex workers visiting a drop in centre in Mombasa County, Kenya
Source: PLOS Glob Public Health. 2022 Nov 11;2(11):e0001247. doi: 10.1371/journal.pgph.0001247 (PMC10045590; doi:10.1371/journal.pgph.0001247)
Supplement: Attachment — Submitted filename: Response to Reviewers.docx [file pgph.0001247.s002.docx]

**Department of Environment and Health Sciences**

| **Personal Tel**  **+254-722 822 448** |  |  | **P.O Box 90420-80100, Mombasa.**  **E-mail:** : valbudambula@gmail.com |
| --- | --- | --- | --- |

**Date**: 4^th^ May 2022

The Editor

PLOS Global Public Health

**RE: Poly drug use among female and male commercial sex workers visiting a drop in centre in Mombasa County, Kenya**

Thank you for your comments as well as reviewers comments. We have responded accordingly as outlined below:

1. Please amend your Financial Disclosure statement. If you did not receive any funding for this study, please simply state: “The authors received no specific funding for this work.” **√ (Page 13, line 397)**

2. Please update your Competing Interests statement. If you have no competing interests to declare, please state: “The authors have declared that no competing interests exist.” **√(Page 13, line 399)**

3. In the online submission form, you indicated that “The data sets analyzed during the current study are available from the corresponding author on reasonable request.”. All PLOS journals now require all data underlying the findings described in their manuscript to be freely available to other researchers, either 1. In a public repository, 2. Within the manuscript itself, or 3. Uploaded as supplementary information.This policy applies to all data except where public deposition would breach compliance with the protocol approved by your research ethics board. If your data cannot be made publicly available for ethical or legal reasons (e.g., public availability would compromise patient privacy), please explain your reasons by return email and your exemption request will be escalated to the editor for approval. Your exemption request will be handled independently and will not hold up the peer review process, but will need to be resolved should your manuscript be accepted for publication. One of the Editorial team will then be in touch if there are any issues. **√** **(Uploaded as supplementary information)**

4. Please provide separate figure files in .tif or .eps format only and remove any figures embedded in your manuscript file. **√ (done)**

Reviewer #1: Dear Authors

It was found difficult to review, when there is neither page no. nor any line numbers, so pls assign pagination and line numbers so it would be easier for the reviewers. **Pagination assigned** **√.**

1. The manuscript is not in Vancouver style **√** **(these changes have been effected throughout the document)**
2. In introduction last sentence, do you wish to check the hypothesis, if so, I could not find addressed in results section addressing it. **√** (**This line has since been deleted. Introduction section, page 4, last paragraph)**
3. Sample size has been applied with FPC. I recommend to report the value of n/N, so that the reader could assess whether it is applicable or not? **√** **The value of n has been elaborately described on page 4, lines 104 t0 111. However, it is not possible to not possible to estimate N as the population of sex workers remains unknown since it is a hidden population.**
4. Table 3 and 4, it is not clear that what the p-values signifies? I mean, the groups compared are not clear? So, better recommended to clarify and re-write. **We have explained that Chi-square or Fisher’s exact test were used to compare distribution of the drugs between or across the demographic features because the sample size was not powered to identify risk factors for individual drug (through formal regression models), Lines 122 to 124. We have also indicted the test used to generate the P-values in the results text and added the actual P-values on Tables 3 and 4.**
5. In table, legend * is not clearly explained. **In Tables 3 & 4, indicates significant difference (P<0.05). We have a footnote on the table to explain this. In table 5, * indicates total percentage >100% because participants can have more than one response. This is explained on the footnote.**
6. Paragraphs, 3 and 5, in results section, the p-values reported and cited in corresponding tables do not possess the exact p-values, so clarify and recommended to re-write. **As stated above in previous response, we have added the actual P-values in the tables.**
7. In same, paragraph 6, the kappa values were not found in any tables? Might be needed.

**Thanks for pointing this out. We have added a new table 5 with the kappa coefficients and level of agreement.**

8. In discussion, 2nd paragraph (about the sex trade among college youth) and last paragraph (further recommendation of other drugs), recheck and clarify.

Paragraph 2: **In this study the whole sample reported attainment of formal education with the majority (85%) having attained post primary education. This is a deviation from most studies among commercial sex workers (CSW) and drug users in Kenya and elsewhere. These two sub-populations tend to report extremely low levels of education. Most of the participants were in colleges or had attained college education and therefore we attempted to link college youth and sex work. This is well documented in cities** **like Nairobi, Kenya.**

Last paragraph (further recommendation of other drugs), recheck and clarify. **This section has been moved to conclusion and clarified (lines 363-365).**

Reviewer #2:

1. The discussion should be done to reflect the findings from the study. **We appreciate and respect this observation. We have gone over and over and we believe to the best of our knowledge our discussion is in line with study findings. We wish the reviewer could be more specific.**
2. Need to look at relationships among the variables. **Association of reported and saliva tested drugs with demographical features were tested using chi-square test of association or Fishers’ exact test (where N per cell was <5) because the sample size was not powered to indefinite risk factors for individual drugs. This has been clarified in lines 121-124.**
3. Also there are quite some grammar errors especially at the discussion section. The corrections have been effected throughout the document. **For example the self-reported in hyphenated.**
4. An example of these is the third sentence of the first paragraph of the discussion section. **This could have been due to different word versions. We will also attach a pdf version.**
5. Some results were presented in the discussion section. **We appreciate and respect this observation. We have gone over and over and this is not the case. We wish the reviewer could be more specific by pointing out paragraphs that need to be corrected.**

Please do not hesitate to contact me in case of any further clarification. I submit.

Sincerely,

Valentine Budambula, PhD
