# Supplementary material for: Poly-drug use among female and male commercial sex workers visiting a drop in centre in Mombasa County, Kenya
Source: PLOS Glob Public Health. 2022 Nov 11;2(11):e0001247. doi: 10.1371/journal.pgph.0001247 (PMC10045590; doi:10.1371/journal.pgph.0001247)
Supplement: Attachment — Submitted filename: RESPONSE TO REVIEWERS.docx [file pgph.0001247.s003.docx]

**Department of Environment and Health Sciences**

| **Personal Tel**  **+254-722 822 448** |  |  | **P.O Box 90420-80100, Mombasa.**  **E-mail:** : valbudambula@gmail.com |
| --- | --- | --- | --- |

**Date**: 13^th^ Sept 2022

The Editor

PLOS Global Public Health

**RE: Revised Manuscript- Poly drug use among female and male commercial sex workers visiting a drop in centre in Mombasa County, Kenya**

On behalf of my co-authors I wish to thank you for accepting our manuscript. The following changes have been made

# Review Comments to the Author

1 Reviewer #2: Please remove this from the discussion section because it is not part of the discussion "Some of the respondents reported to be currently using rohypnol (7.3%) and diazepam (4.1%). This was strengthened by 7.7% of the participants testing positive for benzodiazepines". The implication of the above results should be discussed. Well noted, changes have been implemented - Line 382
2 In the results section, line 204 where you report ‘Nearly all the respondents (98%) had concurrent……’, it is not correct. Recalculate. Has been corrected to “Nearly all the respondents (N=222, 99.11%) had more than one sexual partners with 93.75% reporting three or more sexual partners.” Line 204 to 206
3 In Table 1: The decimal places are not given when reporting proportions in the brackets. Table 1 has been corrected and updated.
4 In the results section line 211, it is not reported what happened to the 4 participants? Did they decline to answer the questions on drug use or they did not report any history of drug use? It is not clear. Apologies. This was an oversight. Corrected statement reads “Of the 224 study participants, 220 (98%) reported current use of drugs while the remaining four (1.79%) were currently not using drugs” line 211-2.
5 Fig 1A: You have reported ‘Miraa’ but in the text it is not mentioned anywhere, have that translated or use the standard term as in the text. Miraa is a local name for khat. We have changed to khat to align with term used in the text. Please refer to corrected attached document (Figure A).
6 In the result section, line 168 , line 273, line 363 have grammatical errors. We have tried our best to look for the grammatical errors to no avail. We wish the reviewer would point out the exact errors.

# Journal Requirements:

1. We have noticed that you have uploaded Supporting Information files, but you have not included a list of legends. Please add a full list of legends for your Supporting Information files after the references list. Added: Page 27, line 682
2. Additional Editor Comments
a) Standardize the reporting of decimal points: The decimal places have been reported to two digits now apart from P-values which are reported as they are.
b) It would be helpful to adjust for sampling weights and clustering. The sample size estimation was misplaced as it did not account for design effect. Response: The study population is sexual commercial workers aged above 18 years accessing services at Mvita Drop in Service Centre, Mombasa County (one centre). It was not a community-based study. As such no clusters were involved. Therefore, adjusting for sampling weights and clustering is not applicable.
c) It would have been helpful to determine the sensitivity and specificity of self-reported drug use information against the saliva assays. Response: This point is well noted however, the objective of the study was to calculate the drug agreement and not sensitivity or specificity. We have reported the Kappa agreement coefficient in Table 5. Sensitivity and specificity are different questions that we are answering in a separate manuscript therefore it will not be prudent to overlay the content of the two manuscripts.
d) The analysis is descriptive – I wonder whether authors would be keen to carry out additional analyses. For example, regression? Response: Thank editor for this comment and suggestion of using regression. This paper by design is descriptive, where we conducted comparison we used univariate approach like chi-square tests in tables 3 & 4. We felt including regression analysis in this descriptive paper would blur the message. Because we have at least seven drugs, using these drugs as the dependent variables, we would produce at least 14 regression models (7 for reported and 7 for saliva confirmed). However, we are using a different approach where we will group commonly used drugs into classes using latent class analysis and later use appropriate regression model that can take dependent variable with more than two levels (binary). This will be a separate manuscript. We therefore, would not want to create overlaps by adding any regression analysis to this manuscript.

Please do not hesitate to contact me in case of any clarification. I submit.

Sincerely,

Valentine Budambula, PhD
